# Candidate Genes for the Diagnosis and Treatment of Alzheimer's Disease in People with Down Syndrome: Innovative Perspectives from Horizon Scanning

**DOI:** 10.1002/alz70855_103606

**Published:** 2025-12-24

**Authors:** Marina Nascimento Silva, Augusto Afonso Guerra‐Júnior

**Affiliations:** ^1^ Federal University of Minas Gerais, Belo Horizonte, Minas Gerais, Brazil

## Abstract

**Background:**

Down syndrome (DS), caused by trisomy of chromosome 21, increases the risk of developing Alzheimer's disease (AD), a prevalent neurodegenerative condition. The presence of an extra copy of chromosome 21 contributes to AD pathophysiology due to the overexpression of genes such as the amyloid precursor protein (APP) gene. As individuals with DS live longer, understanding genetic factors linked to AD is critical for advancing diagnostic and therapeutic approaches. This study investigated candidate genes for AD diagnosis and treatment and explored their potential through Horizon Scanning in health technology assessment (HTA).

**Method:**

A systematic review was conducted in Medline and Embase databases. From 370 retrieved articles, 94 were selected for full‐text review, and six were included in the final analysis.

**Results:**

The analysis identified 11 genes (in addition to APP) directly or indirectly involved in mechanisms related to the pathophysiology of AD in individuals with DS. These include genes associated with APP processing (APOE, BACE2, DYRK1A, SYNJ1, PICALM, CALHM1), tau protein post‐translational modification (RCAN1 and MARK4), neuroinflammation (S100β and RCAN1), oxidative stress (SOD1), and β‐amyloid peptide clearance (CST3). Other genes, such as COL18A1, IDE, and SORCS1, were also highlighted, although their roles in AD pathophysiology remain unclear. Notably, some genes are involved in multiple mechanisms. For instance, DYRK1A and SYNJ1, both located on chromosome 21, are implicated in APP processing and neuroinflammation. DYRK1A overexpression is associated with tau hyperphosphorylation, leading to the accumulation of neurofibrillary tangles. Additionally, DYRK1A increases APP phosphorylation, facilitating its cleavage and subsequent accumulation of β‐amyloid peptides, which aggregate into β‐amyloid plaques. DYRK1A has also been suggested to play a role in neuroinflammation, according to preclinical studies.

**Conclusion:**

From a Horizon Scanning perspective, the identified genes have significant therapeutic potential for AD in DS. DYRK1A inhibitors have shown promise in preclinical studies by reducing cognitive decline and slowing disease progression. Ongoing clinical trials with non‐competitive inhibitors of DYRK1A further support its relevance. Additionally, SYNJ1, SOD1, and RCAN1 are promising inflammatory markers for early AD diagnosis. Research on DYRK1A inhibitors and gene‐specific therapies could drive the development of innovative diagnostic and therapeutic strategies for AD in DS and the broader population.

